# Seasonal roost selection and activity of a remnant population of northern myotis in Pennsylvania

**DOI:** 10.1371/journal.pone.0270478

**Published:** 2022-07-01

**Authors:** Mattea A. Lewis, Gregory G. Turner, Michael R. Scafini, Joseph S. Johnson

**Affiliations:** 1 Department of Biological Sciences, Ohio University, Athens, Ohio, United States of America; 2 Pennsylvania Game Commission, Harrisburg, Pennsylvania, United States of America; University of Western Ontario, CANADA

## Abstract

The decline in northern myotis (*Myotis septentrionalis*) populations due to the disease white-nose syndrome (WNS) has led to the species receiving federal protection in the United States and Canada, requiring conservation of critical habitats. However, considerably more is known about summer habitat preferences of northern myotis compared to late summer through winter. Our goal was to describe the seasonal presence and habitat use of a remnant colony of northern myotis in central Pennsylvania. We radio-tagged 31 northern myotis and established 6 acoustic monitoring stations to document activity from 2017–2021. We found that roost trees used during the maternity season by reproductive females were occupied by bats during both summer (21 June–14 August) and autumn (15 August–31 October), indicating similar habitat use patterns between seasons. During this time, both males and females preferred to roost in dead and declining trees. No other variable influenced male use, but females also preferred trees located close to water and in forest stands with higher basal area than randomly located trees. Northern myotis with active transmitters never left the study area and were tracked to roosts until early November. During October and November, a female and male were tracked to an underground network of air-filled voids (the Milieu Souterrain Superficiel) we presume to be a hibernaculum. Northern myotis calls were recorded outside this roost between March and October, and bats were observed emerging from this roost during spring and autumn but not summer. Acoustic activity at this site exhibited a seasonal pattern that differed from acoustic activity near roost trees and foraging areas, with a peak of activity during late summer when northern myotis are known to swarm. These data show that northern myotis maternity roosts are used extensively outside of summer and may be vulnerable to forestry practices that occur even outside of the pup-rearing season. These data also support the growing evidence that some northern myotis hibernate outside of caves and mines.

## Introduction

Several of the most abundant bat species in eastern North America have declined precipitously since the introduction of the fungus that causes white-nose syndrome (WNS) to the continent. Bats with WNS were first observed in New York state in 2006 [1] and the disease has since spread west to California, south to Texas, and north into Canada. This disease disrupts the physiology of bats during hibernation [2–4] and leads to mass mortality in several species that over-winter in caves [5, 6]. This crisis has brought about a renewed focus on studying bats so that remnant populations can be protected and managed to promote their recovery [7]. For some bat species, investigating seasonal transitions between summer and winter habitats may provide valuable insight into previously unrecognized but important habitats [8, 9] or possible refugia [10].

Several WNS-susceptible species form maternity colonies during the summer months. Maternity colonies can help reduce the cost of thermoregulation [11] and provide opportunities for social interactions and information transfer [12, 13]. These benefits may decline once young are weaned, leading females to change their patterns of habitat use after the lactation period [14, 15]. Maternity colonies progressively dissolve during this time [16–18], after which our knowledge of ecology declines sharply for most species. It is broadly known that many cave-hibernators swarm following the end of the maternity season. During the swarm, bats are thought to evaluate potential hibernacula while spending the day in roosts such as trees, buildings, and rock crevices outside of hibernacula [19, 20]. Although swarming is well-known to include a noticeable increase in activity at hibernacula [21], little is known about the roosts used by bats during this time. This knowledge gap may hinder management of seasonally important habitats, a matter of increasing importance as species receive protected conservation status.

The northern myotis (*Myotis septentrionalis*) was considered amongst the most common and widespread bats in eastern North America based on summer capture rates prior to WNS [22] but is now listed as threatened in the United States and endangered in Canada. The severity of the threat posed by WNS to northern myotis is considered extreme based on winter counts of hibernating bats [5], a result mirrored in declining summer captures [23, 24]. However, northern myotis have long been suspected to hibernate in structures other than caves and mines [25]. Habitats recently identified as winter roosts include crevices within large rock faces in Nebraska [8] and trees in the Mid-Atlantic Coastal Plan [10]. In the Coastal Plain, forests were used by northern myotis year-round, suggesting at least some members of the population were non-migratory. Sedentary populations of northern myotis were first reported by Griffin (1945), who recovered several banded bats < 8 km from their hibernacula, although longer-distance movements are also reported [26].

Remnant populations of northern myotis that are sedentary may represent valuable opportunities for conservation because unlike migratory populations, a management plan developed for a single property can positively affect bats year-round if seasonal habitats are properly identified and managed. During summer, female northern myotis form fission-fusion social groups that primarily roost in trees [27, 28]. Northern myotis appear flexible in their roost requirements, using cavities, crevices, and spaces underneath exfoliating bark of both live and dead trees [29, 30]. Although flexible, female northern myotis consistently select roosts that differ from randomly sampled trees, with important habitat features varying regionally. For example, northern myotis preferred smaller diameter trees in West Virginia [31] and larger trees in New Hampshire [32]. Similarly, northern myotis roosted in areas with greater canopy cover in Illinois [33] and in areas with less cover in Nova Scotia [15]. Roost selection can also differ between sexes, as seen in Arkansas, where female northern myotis roosted in forests with more open canopies than males [29]. Evidence for a seasonal change in roosts used by northern myotis was reported in Nova Scotia, where bats roosted in taller trees surrounded by relatively open canopies during lactation than afterwards [15]. Changing roost selection by females may reflect reduced energetic demands after lactation and may also be influenced by the start of the swarming season, which can last from June through October [34]. Thus, trends in roost use across seasons should be examined to ensure all important habitats for this species are properly managed and protected.

The goal of this study was to describe day roosts used by male and female northern myotis from mid-summer through autumn, to compare roosts used by male and female bats, and to determine if daytime habitat use by females changes after the breakup of maternity colonies. We also sought to determine if the population was sedentary and to locate hibernacula. Based on previous studies reporting seasonal differences in habitat use by females, we hypothesized that maternity roosts would 1) differ from randomly sampled trees, 2) differ from trees used by males, and 3) be abandoned at the end of lactation period.

## Materials and methods

### Study area

**Fig 1:**
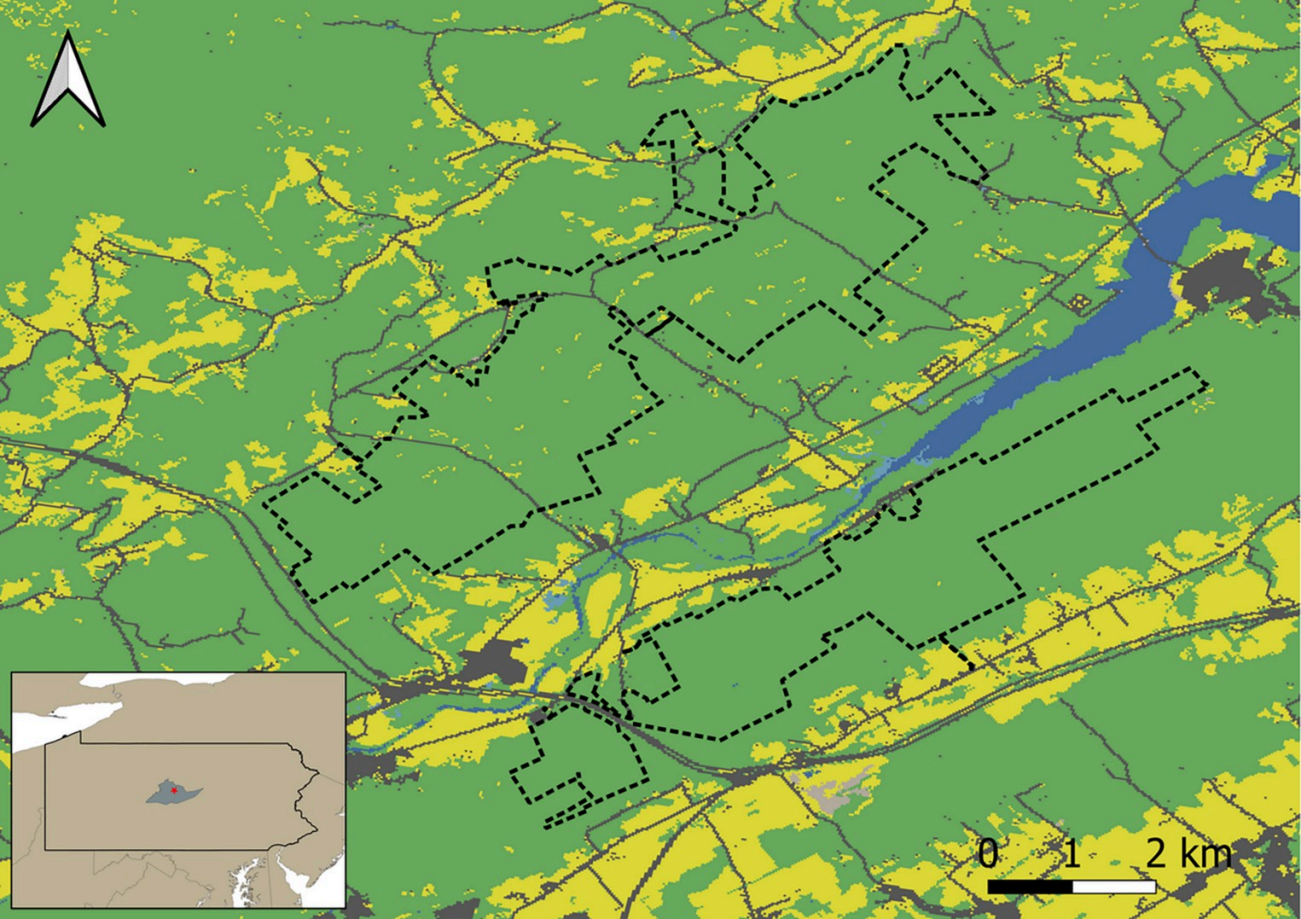
Study area. Map of our study area located in Pennsylvania State Game Lands 92 and 323 (enclosed with dotted boundaries) in Centre County, Pennsylvania. Forest areas are shown in green, pastures and agricultural areas in yellow, water in blue, and developed areas (including roads) in gray. Map inset at bottom left shows the location of Centre County (shaded gray) and the study area (red asterisk) within Pennsylvania.

Our study occurred on Pennsylvania State Game Lands 92 and 323 in Centre County, Pennsylvania (Fig 1). This area is found at the convergence of two physiographic provinces: the eastern section of the Allegheny Plateau and the west end of the Appalachian Ridge and Valley [35]. We treated these properties as single study area due to their proximity, located on either side of Bald Eagle Valley. Bald Eagle Valley contains a small floodplain surrounding Bald Eagle Creek, found upstream of Foster Sayers Joseph Dam, which was created by the Army Corps of Engineers in 1969. The game lands occupying the ridges surrounding Bald Eagle Creek are heavily forested and range from 190 to 502 m in elevation. State Game Lands 323, east of Bald Eagle Creek, is 1201 ha in size while Game Lands 92 on the west side consists of 2136 ha. Agricultural areas and rural developments surround these publicly owned forests that, prior to becoming public land, were used to fuel the local ironworks in the 19^th^ Century and for timber in the 20^th^ Century. Today, both game lands are dominated by oak- (*Quercus sp*.) hickory (*Carya sp*.) forests with lesser amounts of elm (*Ulmus sp*.), maple (*Acer sp*.), and white pine (*Pinus strobus*). While the study area is predominantly forested, small fields that serve as food plots for wildlife can be found within their borders along with several large talus slopes along the west side of the ridge found on Game Lands 323.

### Capture and radio-telemetry

All methods were approved by the Institutional Animal Care and Use Committee of Ohio University (17-H-004) and followed the American Society of Mammalogists’ guidelines for the use of wild mammals in research [36]. We captured bats using mist-nets (Avinet Inc, Portland, Maine) over 157 nights between mid-June to late October of 2017–2021. We placed mist-nets over small bodies of water such as puddles and streams in addition to foraging corridors such as roads and game trails. Nets were opened approximately 30 min before sunset and remained open for a minimum of 5 hrs. Each bat captured was identified to species and the sex, age, reproductive condition, mass (g), and right forearm length (mm) were recorded. Mass and forearm length were measured to the nearest tenth of a unit. We tagged 14 male and 17 female northern myotis with radio-transmitters weighing 0.31g (Holohil Systems Ltd.,Carp, Ontario) or 0.29 g (Lotek Wireless, Newmarket, Ontario). Transmitters were placed between the scapula using surgical glue (Perma-Type Inc, Plainville, Connecticut). We did not trim the hair before putting the transmitter in place to allow the glue and transmitter to form a thick mat with the fur. Bats were not banded and were assumed to be different individuals. Bats were tracked to their day roosts each day using a 3-element Yagi antenna and receiver (Lotek Wireless, Newmarket, Ontario) until the radio-transmitter was shed. We recorded the coordinates of all roosts with a handheld global positioning system (Garmin International Inc., Olathe, Kansas) with 3-m accuracy.

### Day roosting habitat

To test our hypothesis that female roosts would differ from those used by males and randomly sampled trees, we measured eight habitat characteristics of roosts and random trees. We measured tree height (*TreeHt*, m) using a laser rangefinder (Nikon Inc, Melville, New York). Tree diameter (*DBH*, cm) was measured at breast height with dbh tape. Canopy coverage (*CanCov*, %) was measured using a convex spherical densiometer (Ben Meadows Company, Janesville, Wisconsin), with measurements taken at all four Cardinal directions at distances of 0, 5.7, and 11.4 m from the tree. These measures were then averaged to include the entire canopy surrounding the tree. We also coded each tree into one of three categorical decay stages (*Decay*): living, declining, and dead. A living tree was defined as one in good health and with no obvious damage such as broken limbs. Declining trees were defined as those with live foliage but were damaged in a way that may create opportunities for roosting bats. We placed trees in this category if they had numerous or large broken limbs, a snapped top, or dieback. All other trees were classified as dead, regardless of the stage of decay. We used a Cruz-all with a factor of 10 to measure the basal area (a measure of stand density) of the surrounding forest (*BA*, m^2^/ha). Finally, elevation (*Elev*, m), distance from trees to the nearest forest edge (*DistEdge*, m), and their distance to water (*DistWater*, m) were measured using ArcMap (Esri, Redlands, California). We sampled 92 random trees by generating random points in ArcMap within the study area and then measured the tree closest to each location. Random trees were not limited by species or diameter because northern myotis roost in a wide range of trees [29, 30, 33].

We used multinomial regression and model selection to determine which variables differed among male roosts, female roosts, and randomly sampled trees. We constructed 39 multinomial models (S1 Table), each containing a unique combination of roost habitat features as predictor variables and roost type (male roost, female roost, or random tree) as the response variable. This included a null model containing no habitat variables. We limited our list of candidate models to those containing at least one variable known to be important for northern myotis. Only tree roosts were used in the analysis. Because emergence counts revealed that roost trees were used during both summer and autumn (see Results), we included all roosts in the analysis, regardless of which season we discovered them during. Prior to analysis, we ensured that variables were not correlated using Pearson’s correlations and tested for multicollinearity among variables with each model by calculating variance inflation factors. We ranked candidate models using Akaike’s Information Criterion for small samples sizes (AICc) and used a generalized Hosmer–Lemeshow goodness of fit test [37] and the area under the curve receiving operating characteristics [38] to assess the fit of the top model. Multinomial regression models were run in R using the package nnet [39] and AICc comparisons were made using the package MuMIn [40].

### Seasonal use of roosts

We counted bats exiting 47 female roost trees on 121 evenings (2.6 ± 0.2 SE counts per roost) during summer and autumn to describe seasonal use by northern myotis. Counts were conducted when tagged bats were not present, began 30 min before sunset, and continued until it became too dark to see exiting bats. We used a thermal monocular (Scout III 640, FLIR Systems, Inc., Wilsonville, OR, USA) to observe behavior of bats emerging from a rock we suspected was a hibernaculum. To test our hypothesis that maternity roosts would be abandoned during autumn, we used a chi-square test for homogeneity of proportions. This determined if the proportion of nights that maternity roosts were used (e.g., those with emergence counts > 0) declined with the start of autumn. For this analysis, maternity roosts (*n* = 30) were defined as those located from 21 June–14 August. Roosts located from 15 August–1 November were classified as autumn roosts (*n* = 17). We chose 15 August as the start of autumn based upon studies of northern myotis from similar latitudes [34, 41], studies of other *Myotis* species [17], and captures of northern myotis juveniles beginning on 5 August during this study. We did not analyze seasonal change in use of autumn roosts due to small sample size. However, the proportion of autumn roosts used during summer is presented for qualitative examination.

To further investigate seasonal changes in roost use by northern myotis, we modeled the number of bats seen during emergence counts as a function of roost tree habitat and day of the year. We constructed 9 generalized linear mixed models, all with the number of bats as the dependent variable (S2 Table). Fixed effects included day of the year (*Day*) and roost habitat variables found to be important for day roost selection in our multinomial regression models (see Results). Roost identity was used as a random effect. Models were run in the R package glmmTMB [42] with a Poisson distribution. We standardized all factors prior to analysis, checked for correlation between variables using Pearson’s Correlation, tested for multicollinearity among variables by calculating VIFs, and ranked models using AICc. We determined the amount of variation explained by models with ΔAICc < 2 by calculating both the marginal R^2^ (variance explained by fixed effects only) and conditional R^2^ (variance explained by both fixed and random effects).

### Acoustic activity

We deployed 6 acoustic bat detectors (SM4BAT FS, Wildlife Acoustics, Maynard, Massachusetts) within the study area from 6 February through 5 December, 2020 to monitor seasonal changes in activity of northern myotis in the study area. Detectors were deployed in three forest stands in areas where northern myotis were captured during the summer (hereafter, foraging corridors 1–3), in the forest interior at a location where several maternity roosts were found (hereafter, summer roosts), along the edge of a talus slope, and outside a rock roost we located in autumn and suspected to be a hibernaculum. Detectors were programmed to record bat activity beginning 30 min before sunset until 30 min after sunrise. Additionally, detectors were programmed with the High Filter set to 16 kHz, the Sampling Rate set to 256 kHz, a Minimum Duration of 1.5 milliseconds, no Maximum Duration, and the Minimum Trigger Frequency set to 16 kHz.

Recorded call sequences were automatically identified to species using the NY-PA-WV regional classifier within the Northeast Suite of Sonobat v. 4.2.1. Sequences identified as northern myotis were then manually vetted to ensure proper identification. Call sequences identified by the classifier as northern myotis but lacked diagnostic characteristics of a *Myotis* or had obvious features that caused a misidentification were reclassified as unknown. All other sequences identified as northern myotis were retained. We also examined all sequences with ≥ 5 call pulses identified to any species of *Myotis* and classified them as northern myotis if the maximum frequency of any pulse reached 125 kHz. We used a generalized additive model to determine if bat activity varied by ordinal day, using a smoothed function, and among sites using the package mgcv for R [43]. Only vetted northern myotis calls were used in the analysis. Daily average temperature was initially included as a predictor variable but was not significant (*p* > 0.05) and removed from the model. Temperature data were collected onsite using a weather station (Onset Computer Corp, Bourne, Massachusetts).

## Results

**Fig 2:**
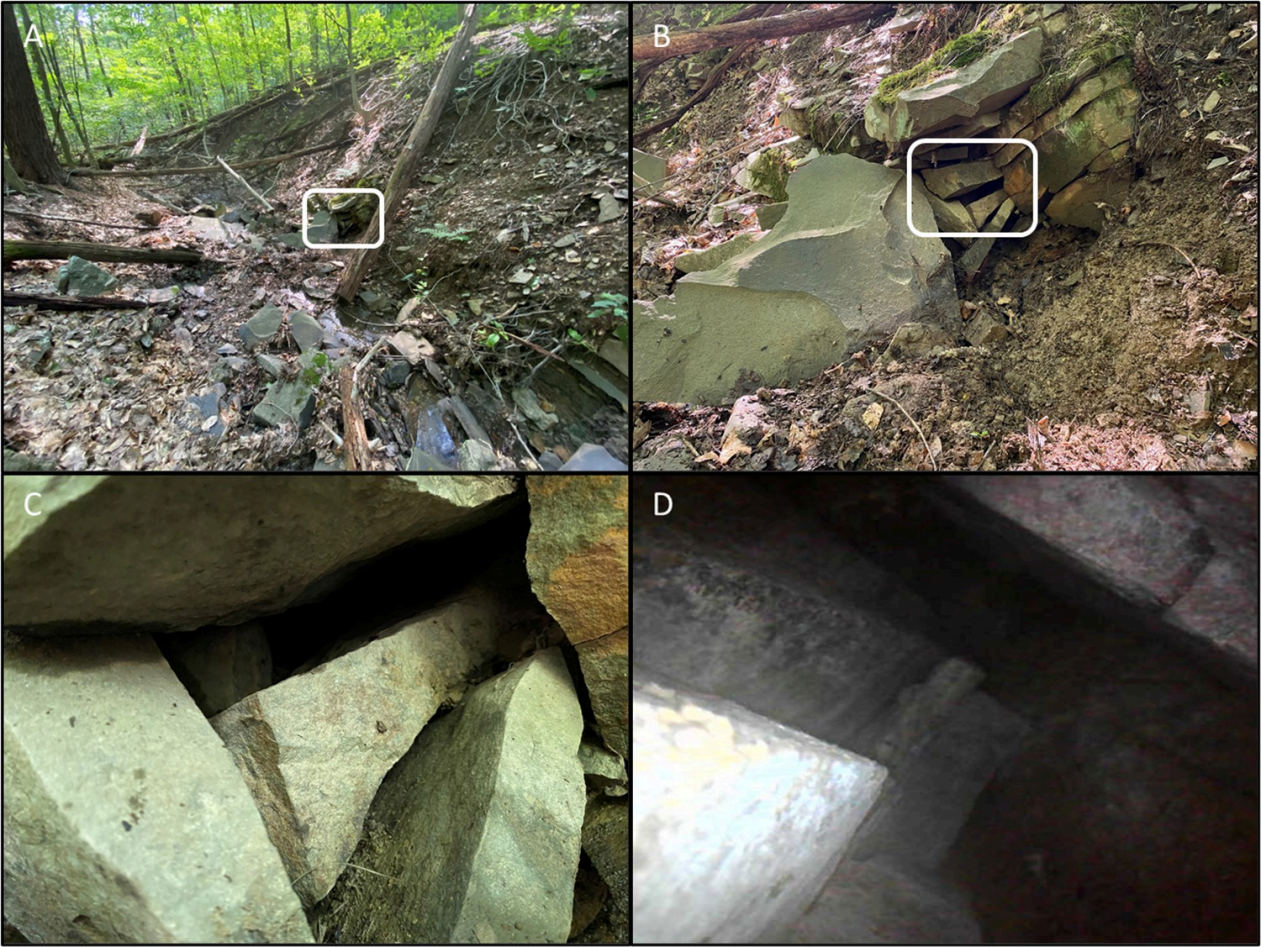
Northern myotis underground roost. Photographs of an underground roost in the Milieu Souterrain Superficiel used by male and female northern myotis between October and March, presumably as a hibernaculum. The white boxes in Panels A and B indicate the areas enlarged in the following panel (Panels B and C). The entrance to hibernaculum was a gap between adjacent rocks that were covered in soil within a narrow drainage. Examination of the main entrance with a borescope found air filled voids extending at least 1 m into the hillside, beyond which we could not explore (D).

We located a total of 69 summer roosts (21 used by male bats and 48 by females) and 20 autumn roosts (12 male, 7 female, and 1 used by both sexes). Of the total 89 roosts, 87 were trees (98%). Radio-tagged bats were tracked to 1–7 trees ($\bar{x}$ = 3 ± 0.3 (SE)) each. One male roosted in the remains of a stone bridge foundation during summer, and both a male and female roosted in an underground network of air-filled voids during October and November, and presumably throughout winter. The underground roost was not located in the talus slope, but instead was found along the bank of a small creek bed, approximately 1.7 m in length, and had multiple entrances in the form of narrow spaces between adjoining rocks (Fig 2). Except for these entrances, the rocks were covered with topsoil. The entrance that bats typically emerged from was approximately 21 cm wide but expanded into the hillside and contained multiple small chambers. We were able to extend a borescope approximately 1 m into the roost but could not maneuver past sharp angles beyond this point, therefore the locations of bats within the roost could not be determined. Emergence counts at this roost found bat use during spring and autumn but not during summer (Table 1). No bat with an active transmitter (i.e., functioning and still glued on to the animal) ever left the study area.

**Table 1:** Summary of emergence counts conducted outside an underground roost we presume to be a hibernaculum for northern myotis in central Pennsylvania.

Month	Number of Counts	Counts with Bats	High Count
February	2	0	0
March	2	1	1
April	2	1	1
May	1	0	0
June	1	0	0
July	6	0	0
August	1	0	0
September	0	–	–
October	5	3	3
November	3	0	0

### Day roosting habitat

**Fig 3:**
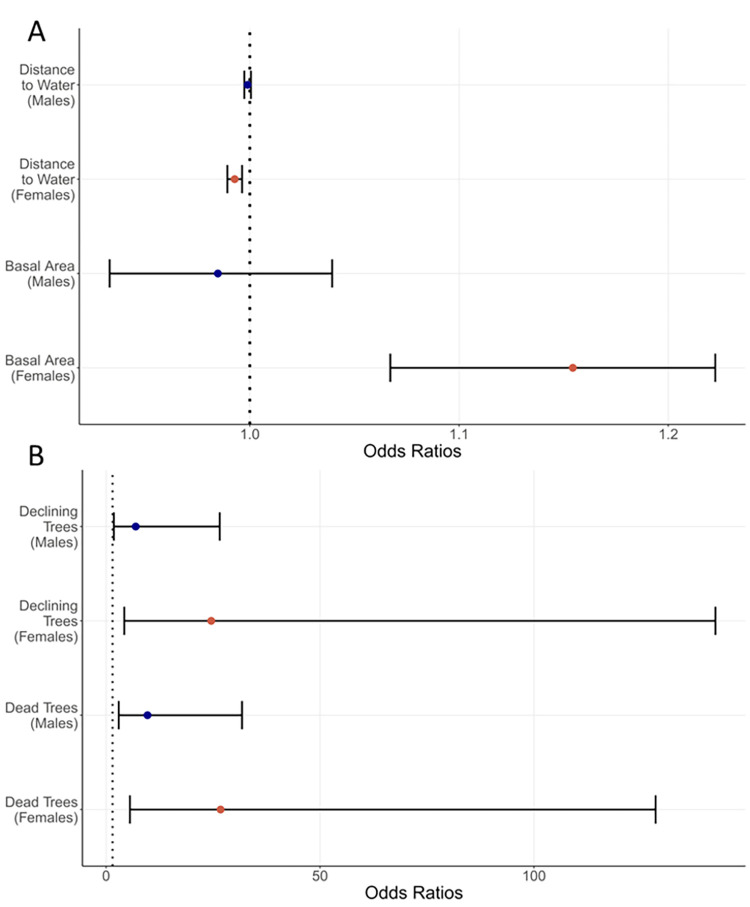
Tree- and stand-scale habitat variables influence the odds of roosting by northern myotis. Odds ratios and 95% confidence intervals for variables included in our top model describing day roost selection by northern myotis in central Pennsylvania. Panel A depicts distance to water and basal area apart from tree decay stage in Panel B to prevent obscuring smaller values. For decay stage, odds ratios are in reference to live trees.

**Fig 4:**
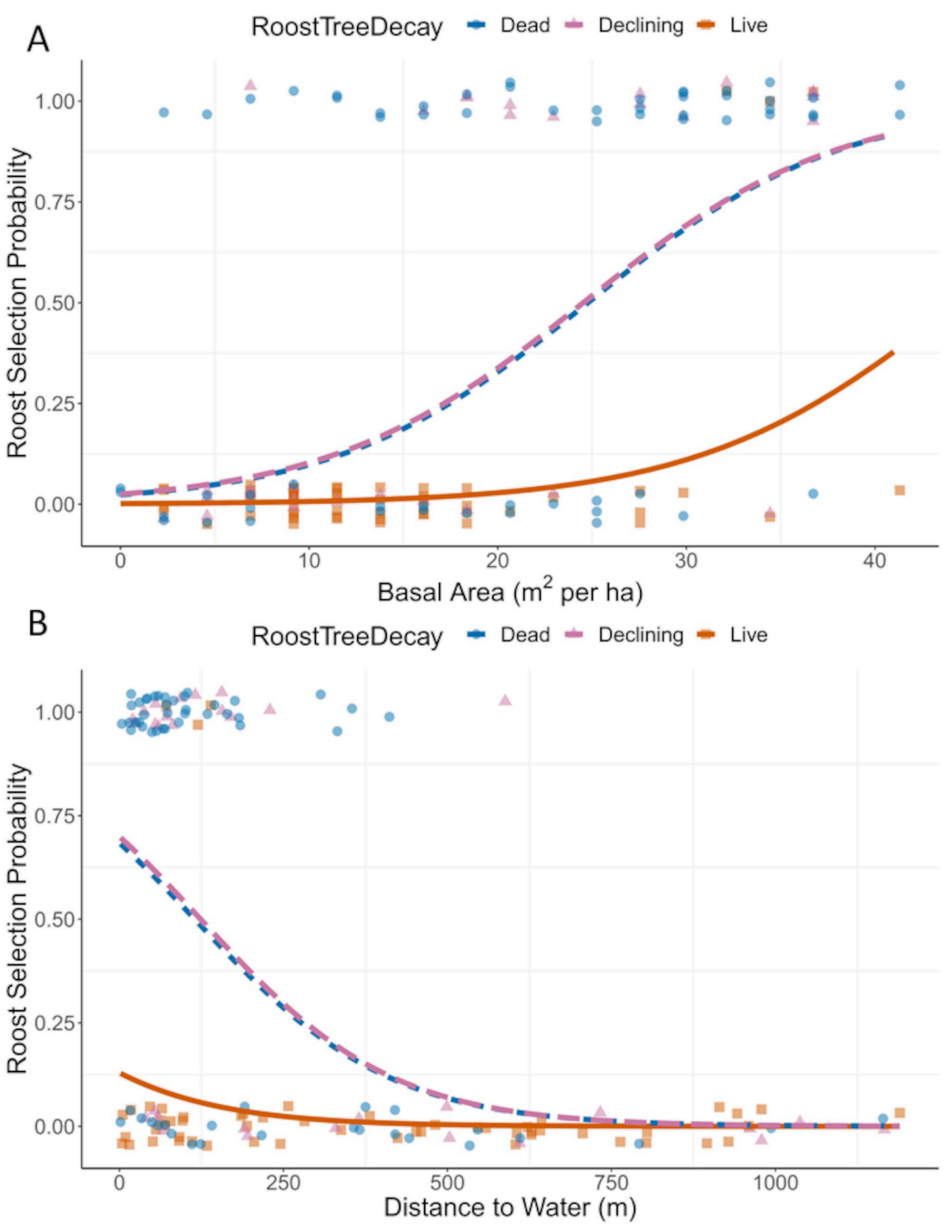
Predictive plots of tree roost use by northern myotis. The probability that trees were used as day roosts by female northern myotis in central Pennsylvania increased with basal area (A) and decreased with increasing distance from water (B), with probabilities changing faster for dead and declining trees than live trees. Basal area and distance to water values for roost (selection probability of 1) and random trees (probability of 0) are plotted with jitter to prevent obscuring repeated values.

Northern myotis roosted in live and dead trees of 16 species (Table 2 and S3 Table) in addition to the rock foundation and underground voids. The model that best predicted roost type (male versus female versus random tree) included stand *BA*, *DistWater*, and *Decay*. No other model had a ΔAICc < 2 (S1 Table). This model accounted for almost 100% of the model weight and had good fit to the data (Hosmer–Lemeshow *X*^2^ = 15.2, df = 18, *p* = 0.64; area under the curve = 0.86). The probability of a tree being used by a female increased by 11% for each unit increase in *BA* (odds ratio = 1.11, CI = 1.07–1.16) but was not significant for males because the confidence interval for the odds ratio crossed 1 (0.99, CI = 0.95–1.03) (Figs 3 and 4).

**Table 2:** Characteristics of roost trees used by male and female northern myotis, along with randomly sampled trees, in central Pennsylvania. Data presented are mean (± SE). See text for descriptions of habitat measures.

Habitat Characteristic	Male Roosts (*n* = 32)	Female Roosts (*n* = 55)	Random Trees (*n* = 92)
*DBH* (cm)	29.3 ± 1.4	28.8 ± 1.5	30.1 ± 1.4
*TreeHt* (m)	16.9 ± 1.1	19.2 ± 0.79	15.2 ± 0.57
*CanCov* (%)	85.0 ± 4.2	57.0 ± 3.1	77.8 ± 2.0
*BA* (m^2^/ha)	11.6 ± 1.2	25.6 ± 1.4	13.5 ± 0.92
*DistEdge* (m)	230 ± 40.9	73.7 ± 11.7	218± 23.2
*DistWater* (m)	284 ± 37.6	111 ± 14.8	399 ± 35.3
*Elev* (m)	320 ± 14.6	218 ± 7.2	316 ± 10.9

Similarly, the probability of a tree being used by female declined by 1% per meter increase in *DistWater* (0.993, CI = 0.989–0.996) but was not significant for males (0.999, CI = 0.997–1.00) (Figs 3 and 4). *Decay* was the only variable that was significant for both sexes. Declining trees were more likely to be used than healthy, live trees by a factor of 7 (odds ratio = 9.7, CI = 1.8–26.6) and 25 (24.6, CI = 4.2–142) for males and females, respectively (Figs 3 and 4). Dead trees were more likely to be used than live trees by a factor of 10 (9.7, odds CI = 2.9–31.8) and 27 (26.7, CI = 5.6–128) for males and females, respectively (Figs 3 and 4).

### Seasonal use of roosts

**Fig 5:**
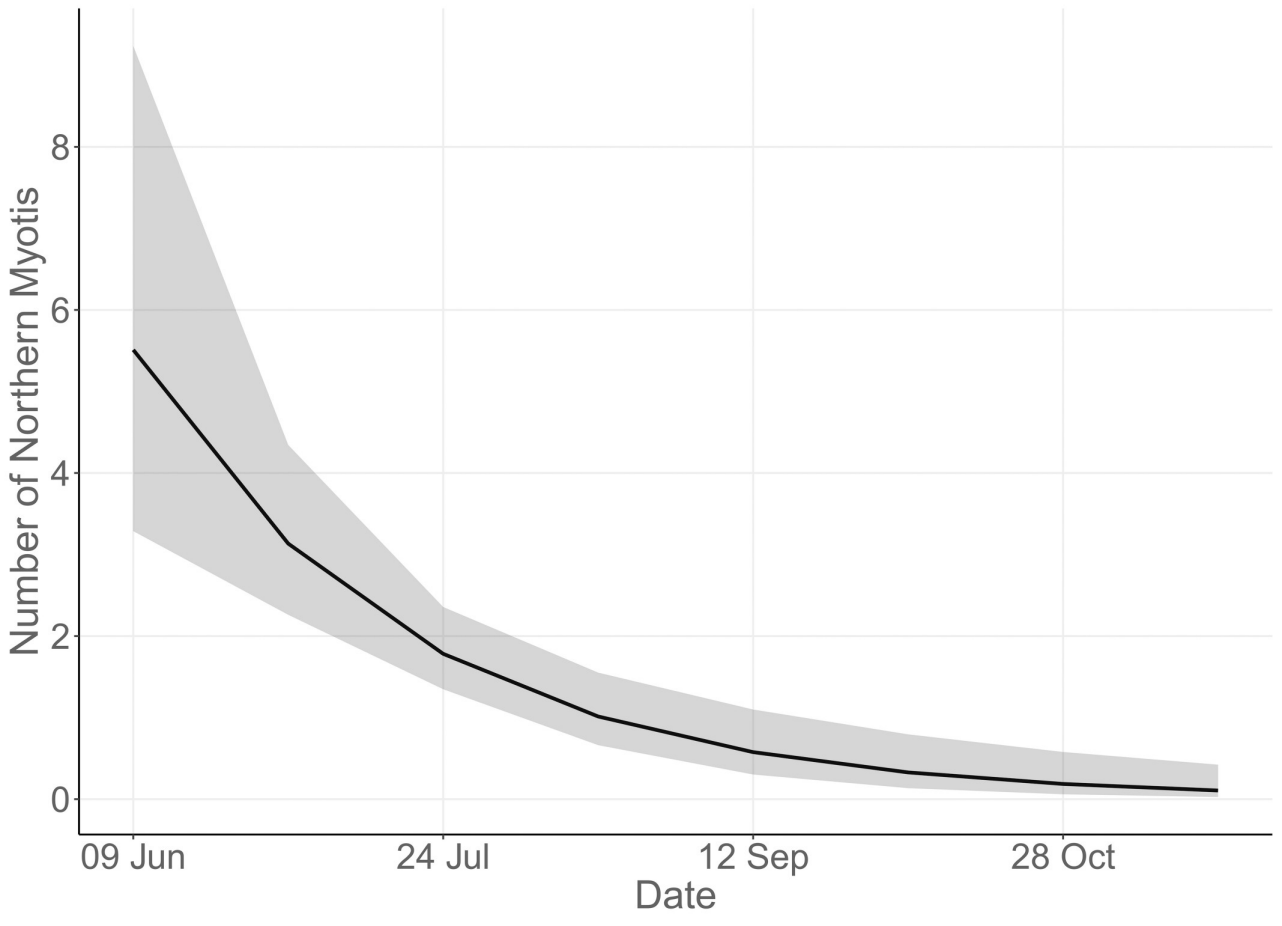
Seasonal trends in northern myotis emergence counts. The number of northern myotis counted exiting roost trees in central Pennsylvania declined as the year progressed (first count occurred on 9 June). Data shown are predicted values with a 95% confidence interval generated from a generalized linear mixed model.

We counted an average of 3.7 ± 0.31 (mean ± SE) bats (range 1–14) emerging from maternity roosts during summer. The proportion of days that maternity roosts were used by bats did not differ between summer (86%, *n* = 32 of 37 trees counted) and autumn (64%, *n* = 14 of 22 trees counted) (χ^2^ = 0.35, df = 1, *p* = 0.55). Although we could not test if the proportion of autumn roosts used differed between seasons, 78% (*n* = 14 of 18 trees counted) of roosts located during autumn were used by bats during summer. The top model predicting the number of northern myotis emerging from roost trees included *Day* as the sole fixed effect (model weight = 0.44). No other model had ΔAICc < 2, although 3 models had ΔAICc values between 2 and 4 (S2 Table). In the top model, counts declined as ordinal day increased (i.e., as the year progressed) (β = -0.56, SE = 0.13, z = -4.3, *p* < 0.001) (Fig 5). Our observations of bats at the rock roost varied seasonally. During spring and autumn, we saw bats emerging from the ground and immediately flying down the drainage and into the forest. We did not observe bats emerging from the roost during the summer, but we did see bats circling the entrance to the roost and flying around the area beginning approximately 30 min after sunset and lasting for over an hour.

### Acoustic activity

**Fig 6:**
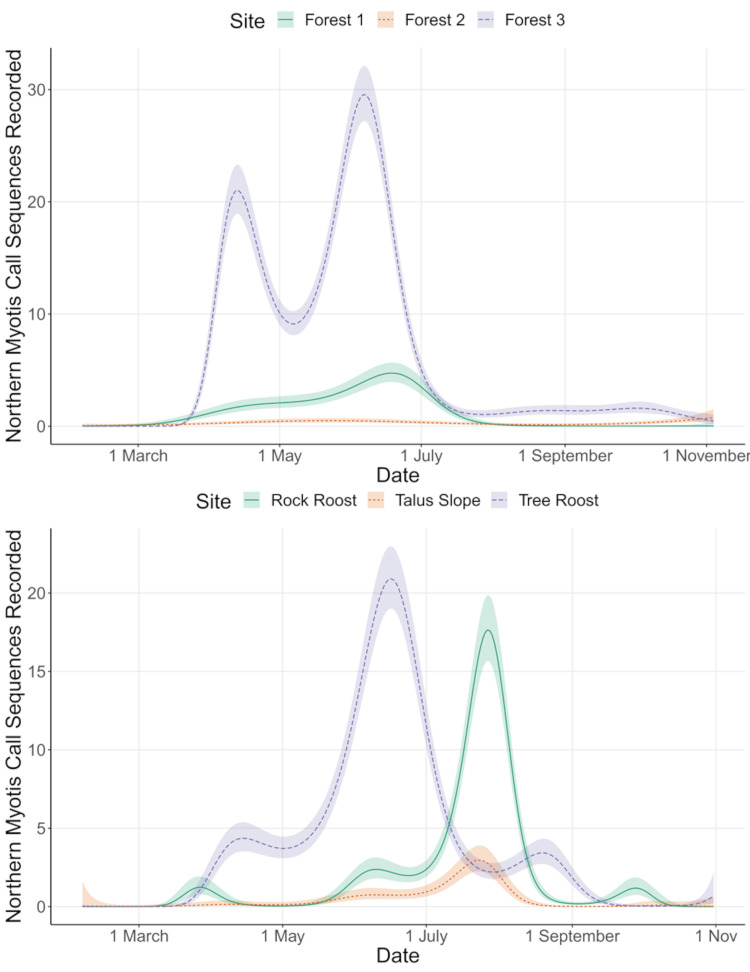
Seasonal trends in nocturnal activity of northern myotis. Acoustic activity of northern myotis recorded at six locations in central Pennsylvania from February–December 2020. Activity recorded at forested corridors where northern myotis were captured (A) and outside maternity roosts (B) peaked earlier in the year than activity outside the rock hibernaculum (B). Data are smoothed as a function of ordinal data and detector site, created using a generalized additive model.

We identified 3,900 northern myotis echolocation call sequences out of 27,703 sequences identified to any *Myotis* species (14.1%) and 179,999 total call sequences (2.2%). The first activity of the year was recorded on 9 March at the rock roost and the last activity was recorded on 6 November at forest corridor 1. The model revealed that the smoothed terms for ordinal day by site were significant for all sampling locations (*p* < 0.05). The deviance explained by the overall model was 61.7%. Examination of the smoothed line for northern myotis activity at the rock roost compared to the foraging corridors showed a peak of activity occurring later in the year (late July and early August) compared to mid to late June at the foraging corridors and summer roosts (Fig 6). A similar but smaller peak in northern myotis activity was recorded at the talus slope in late July and August.

## Discussion

We found that female northern myotis selected roosts from available trees differently than males, and that maternity roosts were used for several months after juveniles were volant and colonies had dissolved. Emergence counts revealed that trees were occupied by progressively fewer bats as summer progressed into autumn but were still used until at least late October when bats began roosting underground in what we presume was a hibernaculum. Acoustic activity outside maternity roosts and in foraging corridors peaked around the time juveniles became volant, followed by steep declines within these habitats and an increase outside of the presumed hibernaculum. The peak of activity at the hibernaculum occurred during August, with lower levels of activity occurring there until November, when activity ceased until early March. These seasonal patterns of activity, along with the absence of bats leaving the study area while radio-tagged, lead us to conclude that some northern myotis in our area were sedentary, making seasonal shifts in habitat use but did not migrate. Our data also provide more evidence that northern myotis do not exclusively hibernate in the caves and mines traditionally considered as hibernacula.

Both male and female northern myotis preferred to roost in trees that were either in decline or fully dead, but we found no other variable that predicted male use of trees. Females, on the other hand, also selected trees that were located close to water and in dense forest stands. Differences in roost selection between male and female bats is not uncommon and may be explained by the greater energetic demands females face during reproduction [44–46]. Previous studies of roost selection in northern myotis have met with widely varying results, indicating that these bats are flexible across their range. Habitat variables such as tree *DBH*, *TreeHT*, and *CanCov* predict roost choice in some studies, but not all, and some studies report contradictory results [16, 29, 32]. However, none of these variables were retained in the top model of day roosting habitat in this study. Instead, *BA* was a significant predictor of female roost use, similar to results from West Virginia [31]. Relatively dense forests may be suitable because they contain many potential roosts or because they provide foraging habitat for northern myotis, which are maneuverable fliers that can glean prey off the surface of vegetation [47, 48]. Distance to water was also a predictor of female roost use, similar to studies of other species [49], possibly due to the increased importance of water to female bats during reproduction [50].

Contrary to our hypothesis, we found that northern myotis occupied summer maternity roosts until November. Thus, we found no support for the hypothesis that females choose roosts with habitat characteristics similar to those used by males after the end of the maternity season [14], unlike observations of northern myotis in Nova Scotia [15]. The number of bats occupying roosts progressively declined throughout late summer and autumn, but use continued through at least 28 October when our last radio-tagged bat was tracked to a tree for the last time. It is unclear how late in the year northern myotis used trees in our area because no emergence counts were conducted after 28 October and our last bat shed its transmitter shortly after this date. However, use of trees during autumn was not unusual, similar to research in Nova Scotia [51], and roosts were just as likely to be occupied during autumn as they were during summer. Northern myotis are known to use trees year-round in coastal North Carolina, where the climate is substantially warmer than that of our study area [10]. In central Pennsylvania, average daily temperatures during late October are often < 10°C, and while minimum temperatures can fall below freezing, average temperatures typically stay above 0°C until mid-November. We confirmed acoustic activity of northern myotis in forest stands until 7 November, demonstrating the ability of this species to be active when bats are often presumed to be hibernating. We therefore urge caution when conducting timber harvests in forests stands known to be occupied by remnant populations of northern myotis in Pennsylvania or regions with warmer winters.

Trees were not the only roosts used by northern myotis. We documented a male roosting in a stone bridge foundation and male and female bats roosted underground during late October and November. Although the bridge foundation was only used by a single bat, the underground roost was used by two radio-tagged bats and multiple bats were seen emerging from this roost during the early spring and throughout the fall. However, no bats were seen emerging from the underground roost during the summer. The peak in acoustic activity that we recorded from late July through early August coincided with visual observations suggestive of swarming behavior, which has been observed beginning in July for northern myotis at a similar latitude in Indiana [52]. We concluded that this site was a hibernaculum based upon this seasonal use and similar observations of northern myotis in Nebraska [8]. The hibernaculum we located is more similar to those used by little brown myotis in Alaska [9]. There, bats hibernated in the Milieu Souterrain Superficiel (MSS), a network of air-filled voids between rocks covered in topsoil [53]. Blejwas and colleagues (2021) suggested that the dispersed nature of MSS hibernacula in Alaska, along with the small sizes of the voids they observed available to roosting bats, might prevent bats from roosting together and potentially slow the spread of WNS. Summer captures of northern myotis in Pennsylvania suggest this species may have declined at a slower rate than two other species vulnerable to WNS, the little brown myotis (*Myotis lucifugus*) and tricolored bat (*Perimyotis subflavus*) [22], lending some support to this hypothesis. However, northern myotis still declined rapidly in Pennsylvania. Thus, while *Pseudogymnoascus destructans*, the fungus that causes WNS, may have taken longer to reach the MSS than traditional hibernacula in Pennsylvania, it is unlikely that the MSS is a refugia from WNS in our area. Nevertheless, non-traditional hibernacula such as this need to be identified for effective conservation. While we cannot conclude talus slopes are used as hibernacula from our data, we recorded a small peak in acoustic activity at the slope concurrent with the peak at the rock roost and encourage further studies of these habitats.

In the years following the arrival of WNS in North America, substantial efforts have been made to understand the disease and enhance the survival of cave-hibernating species. However, it is becoming increasingly apparent that several species susceptible to WNS hibernate outside of caves and mines [8, 20, 54]. Tools developed to mitigate WNS in caves and mines [55] may not always be transferrable to non-traditional hibernacula such as the MSS, highlighting the need to better understand this habitat and the bats that use it. The nature of this subterranean habitat makes it difficult to locate and survey for bats. However, sedentary populations of bats can be followed to these hibernacula using conventional radio-telemetry (Blejwas et al. 2021; this study). These populations represent a unique opportunity for managers because all the year-round critical habitats may be located on lands under a single management authority. In our study, we used radio-telemetry to locate to important roosting and foraging areas, which we then monitored for seasonal trends in activity. This approach led to novel observations about autumn use of maternity roosts and the discovery of a presumed hibernaculum. We recommend others similarly attempt to track summer populations of northern myotis inhabiting areas where MSS habitats are likely to exist to identify priority areas for conservation.

## Supporting information

S1 Table Ranking of all multinomial logistic regression models used to compare northern myotis day roosts and randomly sampled trees in central Pennsylvania. See text for descriptions of variable included in each model. K represents the number of parameters of each model. AICc is the AIC score corrected for small sample sizes. ΔAICc is difference in model AICc score and the score of top model. Weight is the weight of the model.
(DOCX)

S2 Table Ranking of all generalized linear mixed models used to analyze counts of northern myotis emerging from day roosts in central Pennsylvania. See text for descriptions of variable included in each model. K represents the number of parameters of each model. AICc is the AIC score corrected for small sample sizes. ΔAICc is difference in model AICc score and the score of top model. Weight is the weight of the model.
(DOCX)

S3 Table Summary of tree species used by male and female northern myotis in central Pennsylvania, alongside the number of random trees sampled by species. (DOCX)
