# Supplementary material for: Seasonal roost selection and activity of a remnant population of northern myotis in Pennsylvania
Source: PLoS One. 2022 Jul 1;17(7):e0270478. doi: 10.1371/journal.pone.0270478 (PMC9249199; doi:10.1371/journal.pone.0270478)
Supplement: S1 Table — See text for descriptions of variable included in each model. K represents the number of parameters of each model. AICc is the AIC score corrected for small sample sizes. ΔAICc is difference in model AICc score and the score of top model. Weight is the weight of the model. (DOCX) [file pone.0270478.s001.docx]

**S1 Table. Ranking of all multinomial logistic regression models used to compare northern myotis day roosts and randomly sampled trees in Central Pennsylvania.** See text for descriptions of variable included in each model. K represents the number of parameters of each model. AICc is the AIC score corrected for small sample sizes. ΔAICc is difference in model AICc score and the score of top model. Weight is the weight of the model.

| Variables in the Model | K | AICc | ΔAICc | Weight |
| --- | --- | --- | --- | --- |
| *BA + DistWater + Decay* | 10 | 246.46 | 0 | 1 |
| *BA + Decay* | 8 | 270.87 | 24.4 | 0 |
| *DistEdge + CanCov + BA* | 8 | 271.03 | 24.57 | 0 |
| *DistEdge + DistWater + BA* | 8 | 271.68 | 25.21 | 0 |
| *Elev + BA* | 6 | 274.05 | 27.59 | 0 |
| *BA + DistWater* | 6 | 276.57 | 30.1 | 0 |
| *CanCov + Elev + Decay* | 10 | 281.32 | 34.85 | 0 |
| *DistEdge + Decay + CanCov* | 10 | 284.25 | 37.79 | 0 |
| *Decay + Elev* | 8 | 285.3 | 38.84 | 0 |
| *DistWater + Decay* | 8 | 289.19 | 42.73 | 0 |
| *DistEdge + BA* | 6 | 290.29 | 43.82 | 0 |
| *DistEdge + DBH + BA* | 8 | 293.05 | 46.59 | 0 |
| *DistEdge + Decay* | 8 | 301.02 | 54.55 | 0 |
| *DistEdge + Decay + DBH* | 10 | 302.26 | 55.8 | 0 |
| *BA + DBH + TreeHt* | 8 | 305.5 | 59.04 | 0 |
| *DistEdge + CanCov + TreeHt* | 8 | 308.25 | 61.79 | 0 |
| *DistWater + CanCov* | 6 | 308.7 | 62.23 | 0 |
| *BA* | 4 | 312.14 | 65.68 | 0 |
| *DistWater + Elev* | 6 | 312.35 | 65.88 | 0 |
| *DBH + CanCov + DistWater* | 8 | 312.41 | 65.95 | 0 |
| *CanCov + Elev* | 6 | 313.48 | 67.02 | 0 |
| *Elev + TreeHt* | 6 | 313.95 | 67.48 | 0 |
| *DistEdge + DistWater + Elev* | 8 | 314.6 | 68.13 | 0 |
| *BA + DBH* | 6 | 315.91 | 69.45 | 0 |
| *DistEdge + CanCov* | 6 | 318.33 | 71.87 | 0 |
| *DistEdge + DistWater* | 6 | 319.52 | 73.06 | 0 |
| *Elev* | 4 | 321 | 74.53 | 0 |
| *DistEdge + DBH + CanCov* | 8 | 321.9 | 75.44 | 0 |
| *DBH + CanCov + TreeHt* | 8 | 322.68 | 76.22 | 0 |
| *DistEdge + DistWater + DBH* | 8 | 322.93 | 76.47 | 0 |
| *DBH + Elev* | 6 | 323.24 | 76.78 | 0 |
| *DistWater* | 4 | 325.33 | 78.86 | 0 |
| *DBH + DistWater* | 6 | 329.09 | 82.62 | 0 |
| *DBH + CanCov* | 6 | 335.82 | 89.36 | 0 |
| *DistEdge* | 4 | 341.33 | 94.87 | 0 |
| *DistEdge + DBH* | 6 | 344.76 | 98.3 | 0 |
| *DBH + TreeHt* | 6 | 354.96 | 108.5 | 0 |
| *Null* | 2 | 366.53 | 120.07 | 0 |
| *DBH* | 4 | 370.32 | 123.85 | 0 |
