# Supplementary material for: Seasonal roost selection and activity of a remnant population of northern myotis in Pennsylvania
Source: PLoS One. 2022 Jul 1;17(7):e0270478. doi: 10.1371/journal.pone.0270478 (PMC9249199; doi:10.1371/journal.pone.0270478)
Supplement: S3 Table — (DOCX) [file pone.0270478.s003.docx]

**S3 Table. Summary of tree species used by male and female northern myotis in central Pennsylvania, alongside the number of random trees sampled by species.**

| Tree Species | Male Roost Trees | Female Roost Trees | Randomly Sampled Trees |
| --- | --- | --- | --- |
| *Acer rubrum* | 4 | 5 | 7 |
| *Acer saccharum* | 3 | 4 | 8 |
| *Acer saccharinum* | 1 | 2 | 1 |
| *Acer pensylvanicum* | 0 | 0 | 1 |
| *Betula alleghaniensis* | 0 | 0 | 1 |
| *Carya alba* | 0 | 0 | 1 |
| *Carya glabra* | 0 | 0 | 1 |
| *Carya ovata* | 4 | 7 | 2 |
| *Pinus rigida* | 0 | 0 | 2 |
| *Pinus strobus* | 2 | 6 | 8 |
| *Populus grandidentata* | 0 | 0 | 2 |
| *Fagus grandifolia* | 2 | 0 | 9 |
| *Tsuga candanesis* | 1 | 0 | 2 |
| *Fraxinus americana* | 1 | 3 | 2 |
| *Fraxinus pennsylvanica* | 1 | 8 | 1 |
| *Juniperus virginiana* | 0 | 0 | 1 |
| *Juglans nigra* | 0 | 0 | 1 |
| *Liriodendron tulipifera* | 0 | 0 | 6 |
| *Nyssa sylvatica* | 0 | 0 | 3 |
| *Quercus alba* | 3 | 2 | 2 |
| *Quercus coccinea* | 0 | 1 | 1 |
| *Quercus prinus* | 0 | 3 | 8 |
| *Quercus montana* | 2 | 0 | 1 |
| *Quercus rubra* | 2 | 0 | 1 |
| *Quercus velutina* | 1 | 0 | 5 |
| *Robinia pseudoacacia* | 0 | 0 | 1 |
| *Prunus serotina* | 0 | 1 | 2 |
| *Ulmus americana* | 3 | 5 | 2 |
| *Unidentified hardwood snag* | 2 | 7 | 10 |
| Totals | **32** | **54** | **92** |
